# Editorial: Community series in grief disorders: clinical, cultural, and epidemiological aspects, volume II

**DOI:** 10.3389/fpsyt.2023.1259161

**Published:** 2023-08-14

**Authors:** Geert E. Smid, Hannah Comtesse, Clare Killikelly, Birgit Wagner

**Affiliations:** ^1^ARQ National Psychotrauma Centre, Diemen, Netherlands; ^2^University of Humanistic Studies, Utrecht, Netherlands; ^3^Catholic University of Eichstätt-Ingolstadt, Eichstätt, Bavaria, Germany; ^4^Department of Psychiatry, Faculty of Medicine, University of British Columbia, Vancouver, BC, Canada; ^5^Medical School Berlin, Berlin, Germany

**Keywords:** prolonged grief disorder, loss, bereavement, suicide, physician-assisted dying, epidemiology, cultural aspects

The articles included in this Research Topic provide essential insights in clinical, cultural, and epidemiological aspects of grief disorders. Clinical aspects include diagnosis, prevention, and treatment of grief disorders. Cultural aspects involve the provision of care within specific cultural contexts, while dealing with multiculturalism and globalization. Epidemiological aspects encompass both clinical and cultural aspects and include risk for the development or maintenance of disordered grief as well as factors that contribute to resilience and recovery.

## Clinical and cultural aspects

While traumatic bereavement constitutes an established risk factor for prolonged grief disorder (PGD), clinical care aimed at good dying may contribute to the prevention of PGD. The death of a loved one by suicide is among the most frequent causes of traumatic bereavement. Suicide bereavement is associated with an increased risk of mental disorders and suicide in the bereaved (1). Besides suicide, people with a wish to die may consider the option of physician-assisted dying [PAD; (2)]. In a growing number of countries, including the Netherlands, Belgium, Luxembourg, Canada, New Zealand, and Spain, PAD is legally permitted under strict conditions (3); some states in the US have regulations permitting PAD, and in Switzerland, assisted suicide for “selfless reasons” is legally permitted. In some other countries (e.g., Germany), constitutional court rulings have created a defense for physician involvement in PAD, and it is expected that legislation will follow. The topic of PAD is debated worldwide, especially concerning patients with mental disorders. Snijdewind et al. provide insight into grief reactions of suicide bereaved individuals as well as the grief following PAD. Their mixed-methods study is among the first to study grief following both PAD and suicide due to a mental disorder. It clarifies how circumstances or experiences during the period leading up to PAD or suicide of a loved one due to a mental disorder impacts the experienced grief and mental health outcomes of the bereaved partners. They conclude that expectedness of the death of the partner, absence of suffering of the partner at the time of dying, and presence of physician support may in part explain the protective effects they found of PAD against severe grief reactions. Their findings offer empirical insight that can broaden the discussion concerning PAD and suicide by involving grief of bereaved partners into the discussion and may help mental health professionals to reflect on their position in these practices.

As Snijdewind et al. report in this Research Topic, for bereaved life partners, grief following PAD conveys less distress than after suicide. For the social environment of the surviving partner, the fact that a request for PAD had been granted made it easier to understand and accept that the deceased suffered unbearable and irremediable mental suffering. These social factors may contribute to a lower risk of prolonged grief and other mental distress following PAD compared with suicide. However, other sociocultural factors may play a role as well. In the Netherlands, PAD is culturally widely accepted. According to a survey among 3,625 adults living the Netherlands, 87% believe that PAD should be possible in certain circumstances, and 80% support the option of PAD for people with severe mental illness (4). In the absence of broad cultural acceptance, grief following PAD may be less favorable. A study among 85 Swiss relatives or friends who had been present during assisted suicide found more posttraumatic stress disorder and prolonged grief symptoms in bereaved persons who perceived social disapproval of PAD, and perceived disapproval was associated with maintaining secrecy regarding the cause of death (5).

Van Eersel et al. describe a cognitive-behavioral framework to explain the development and maintenance of prolonged grief symptoms following involuntary job loss. These symptoms can lead to additional psychological and practical problems, such as anxiety and depressive symptoms, lower employability, and reduced likelihood of re-employment. Their framework could stimulate more systematic research, as well as the design of tailored interventions for clients suffering from these symptoms.

Groen et al. draw attention to the fact that clinicians sometimes do not relate mental health problems to the loss of a loved one, especially in the case of refugee patients for whom traumatic experiences following war and conflict are at the foreground and whose presentation of symptoms may vary cross-culturally. Their study aimed to assess and compare the prevalence of PGD between non-migrant and refugee patient populations referred for treatment of anxiety, depressive or trauma and stressor-related disorders, and, in addition, to evaluate differences in the prevalence of PGD and cultural aspects related to grief between these groups, while raising awareness and contributing to the development of patient expert assisted psychoeducation of PGD tailored to the patients' needs, norms, and values.

## Epidemiological and cultural aspects

Epidemiological and cultural aspects include the risk of PGD in different cultural contexts and factors associated with PGD risk and resilience. Treml et al. aimed to estimate the prevalence of the new PGD criteria according to the text revision of DSM-5 (DSM-5-TR) in a representative German population-based sample, evaluate the factor structure, sociodemographic, and loss-related correlates of PGD and explore predictors. They found a conditional prevalence of PGD of 3.4%. Confirmatory factor analysis confirmed a unidimensional model of PGD. Regression analysis demonstrated that time since the death, the relationship to the deceased, and unpreparedness for the death were significant predictors of PGD.

As a relatively new disorder, PGD has been investigated in many countries to date. However, relatively few studies have focused on Arabic-speaking populations. As some Arabic-speaking countries are currently struggling with war and Conflict and faced with a lack of mental health resources, it seems important to gather more knowledge on PGD in this population. Specht et al. examined PGD for the first time in a larger sample on Arabic-speaking populations. A substantial proportion of participants, 18.8%, met PGD criteria. Thus, the existing professional support structures in many Arabic-speaking countries may not be sufficient. Their finding that young women in our study were at particularly high risk of developing PGD, and are already a particularly vulnerable group in many countries, underlines the necessity to ensure that adequate treatment options are made available in order to promote personal development, participation in society, and the empowerment of women.

As the articles in this Research Topic show, optimizing the prevention of and care for grief disorders has profound implications for the organization and provision of mental health care, spiritual care, and end-of-life care. This Research Topic exemplifies the important insights that research on different cultural settings and with diverse cultural groups can provide in the field of grief and grief disorders.

## Author contributions

GS: Writing—original draft, review, and editing. HC: Writing—review and editing. CK: Writing—review and editing. BW: Writing—review and editing. All authors approved the submitted version.
